# Development of transgenic *Caenorhabditis elegans* expressing human transthyretin as a model for drug screening

**DOI:** 10.1038/s41598-018-36357-5

**Published:** 2018-12-14

**Authors:** Yukimoto Tsuda, Kunitoshi Yamanaka, Risa Toyoshima, Mitsuharu Ueda, Teruaki Masuda, Yohei Misumi, Teru Ogura, Yukio Ando

**Affiliations:** 1 Department of Neurology, Graduate School of Medical Sciences, Kumamoto University, 1-1-1 Honjo, Chuo-ku, Kumamoto, 860-8556 Japan; 2 Department of Molecular Cell Biology, Institute of Molecular Embryology and Genetics, Kumamoto University, 2-2-1 Honjo, Chuo-ku, Kumamoto, 860-0811 Japan

## Abstract

Familial amyloid polyneuropathy is a hereditary systemic amyloidosis caused by a mutation in the transthyretin (TTR) gene. Amyloid deposits in tissues of patients contain not only full-length TTR but also C-terminal TTR fragments. However, *in vivo* models to evaluate the pathogenicity of TTR fragments have not yet been developed. Here, we generated transgenic *Caenorhabditis elegans* strains expressing several types of TTR fragments or full-length TTR fused to enhanced green fluorescent protein in the body wall muscle cells and analyzed the phenotypes of the worms. The transgenic strain expressing residues 81–127 of TTR, which included the β-strands F and H, formed aggregates and caused defective worm motility and a significantly shortened lifespan compared with other strains. These findings suggest that the C-terminal fragments of TTR may contribute to cytotoxicity of TTR amyloidosis *in vivo*. By using this *C*. *elegans* model system, we found that (−)-epigallocatechin-3-gallate, a major polyphenol in green tea, significantly inhibited the formation of aggregates, the defective motility, and the shortened lifespan caused by residues 81–127 of TTR. These results suggest that our newly developed *C*. *elegans* model system will be useful for *in vivo* pathological analyses of TTR amyloidosis as well as drug screening.

## Introduction

Hereditary transthyretin (ATTRm) amyloidosis, also called transthyretin (TTR)-related familial amyloid polyneuropathy (TTR-FAP), is a fatal inherited disease associated with extracellular amyloid deposits derived from TTR^1^. Patients with ATTRm amyloidosis demonstrate polyneuropathy, autonomic dysfunction, cardiac and renal failure, gastrointestinal dysfunction, and other symptoms, all of which may lead to death usually within 10 years^2^. More than 140 mutations in the TTR gene have now been reported, with the mutation Val30 to Met (Val30Met) being most common and mainly reported in Japan, Portugal, and Sweden^3^.

TTR forms a 55-kDa homotetramer that consists of four identical 14-kDa monomers with 127 amino acid residues. TTR is mainly produced (secreted) in the liver, eye, and choroid plexus, and it usually exists as a tetramer in the bloodstream^4^. In patients, TTR dissociates to monomers that are misfolded by mutations and/or aging, which causes polymerization of the dissociated TTR and formation of amyloid fibrils^5^. Liver transplantation is the most common treatment for patients with ATTRm. Dissociation of the TTR tetramer into monomers is the rate-limiting step in amyloid fibril formation^5^. Therefore, small molecules that can stabilize the TTR tetramer have been developed as therapeutic agents^6^, and diflunisal and tafamidis are now used as TTR stabilizers^7–10^. Although liver transplantation and TTR tetramer stabilizers effectively treat ATTRm amyloidosis, their effects are limited to early stages of the disease and the delay of disease progression, but they do not completely suppress the progression of the pathology^11^.

Involvement of TTR fragments in the formation of amyloid fibrils has been demonstrated. Amyloid deposits in the tissues of ATTR amyloidosis consist of not only full-length TTR but also C-terminal TTR fragments, especially the fragment with residues 49–127 (TTR_49–127_)^12–15^. TTR_49–127_ can be produced by trypsin treatment of full-length TTR, and it induced amyloid formation in *in vitro* studies^16,17^. Structural analysis revealed that the β-strands F and H (residues 91–96 and 115–124, respectively) of TTR had a strong amyloidogenic properties^18^. However, how TTR fragments affect amyloidosis *in vivo* remains elusive.

For many years, many attempts have been made to develop an animal model of TTR amyloidosis to clarify the molecular mechanism of the pathogenesis of this disease and to evaluate the therapeutic effects of candidate drugs^19^. However, transgenic mice and rat models so far reported unfortunately have not manifested the toxic phenotype representing TTR amyloidosis^20,21^. Transgenic worms expressing human disease-relevant proteins and/or peptides have been developed, however, and have provided information about the molecular mechanisms of disease pathogenesis and served as an efficient screening tool for drug development^22–25^. (−)-Epigallocatechin-3-gallate (EGCG) is the major polyphenol in green tea^26^. Reports have described certain biological functions of EGCG such as antioxidant and anti-inflammatory activities^27–30^. EGCG has also been demonstrated to inhibit toxic aggregate formation of Aβ, α-synuclein, ataxin-3, and mutant huntingtin, as well as bacterial amyloid formation^26,28,31–37^. In this study, we describe a *Caenorhabditis elegans* model that expresses various TTR fragments to elucidate the pathogenesis of C-terminal fragments of TTR *in vivo*, and we established this model as a tool for screening drug candidates for TTR amyloidosis.

## Results

### C-terminal fragments of TTR formed aggregates and demonstrated toxic effects *in vivo*

**Figure 1:**
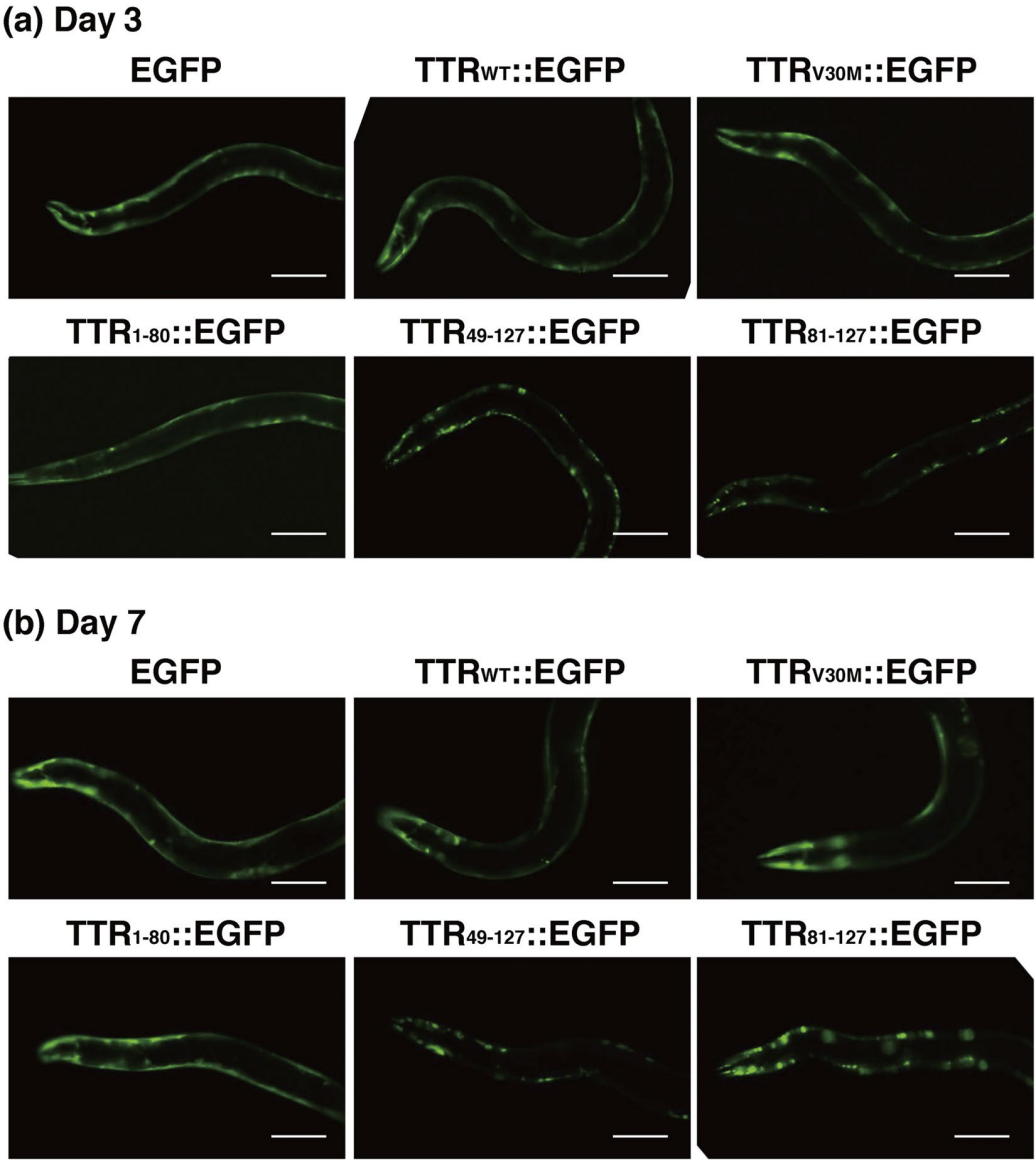
Expression of TTR::EGFP fusion proteins in *C*. *elegans*. Strains expressing EGFP, TTR_WT_::EGFP, TTR_V30M_::EGFP, TTR_1–80_::EGFP, TTR_49–127_::EGFP, and TTR_81–127_::EGFP were analyzed. Young adult (Day 3) (**a**) and aged (Day 7) (**b**) worms of each strain were evaluated. The day when eggs were laid was designated day 0. Scale bar: 100 μm.

To investigate the pathogenesis of C-terminal fragments of TTR *in vivo*, we generated a *C*. *elegans* model expressing various TTR fragments fused to enhanced green fluorescent protein (EGFP): the full-length wild-type TTR (TTR_WT_::EGFP), the 1–80 residue fragment (TTR_1–80_::EGFP), the 49–127 residue fragment (TTR_49–127_::EGFP), the 81–127 residue fragment (TTR_81–127_::EGFP), the full-length TTR but containing a Val30Met mutation (TTR_V30M_::EGFP), and EGFP alone (EGFP) as a control. TTR_49–127_ was previously identified in amyloid deposits obtained from patients^12–15^, and TTR_81–127_ contains two strong amyloidogenic β-strands—F and H^18^. The Val30Met mutation is the most common TTR mutation in the world^3^. Expression of fusion proteins in the body wall muscle cells of *C*. *elegans* was achieved by using the *unc-54* promoter. Note that TTR::EGFP fusion constructs do not contain a TTR signal sequence; as a result, these fusion proteins are expected to localize in the cytoplasm of the body wall muscle cells. As Fig. 1 shows, EGFP and TTR_1–80_::EGFP exhibited a diffuse localization pattern throughout the body wall muscle cells. In contrast, TTR_49–127_::EGFP and TTR_81–127_::EGFP had discrete aggregates. TTR_WT_::EGFP and TTR_V30M_::EGFP, however, gradually formed aggregates as the worms aged (Table 1). Based on fluorescence microscopic findings, these kinds of TTR were likely to form intracellular amorphous shape aggregates in our *C*. *elegans* models. These results suggest that the C-terminal fragments of TTR play an important role in aggregate formation *in vivo* as well as *in vitro*.

**Table 1:** Effect of C-terminal TTR fragments on formation of aggregates.

Strain	Number of aggregates per worm (n = 20)	
	Day 3*	Day 7*
EGFP	1.5 ± 1.6	3.2 ± 1.7
TTR_WT_::EGFP	5.3 ± 3.4	30.5 ± 10.5
TTR_V30M_::EGFP	4.2 ± 1.6	17.4 ± 6.8
TTR_1–80_::EGFP	0.7 ± 1.1	3.5 ± 1.9
TTR_49–127_::EGFP	67.0 ± 21.9	92.5 ± 29.4
TTR_81–127_::EGFP	124.2 ± 21.3	176.1 ± 14.8

*Day 0 = the day when eggs were laid. Data are means ± SD.

**Figure 2:**
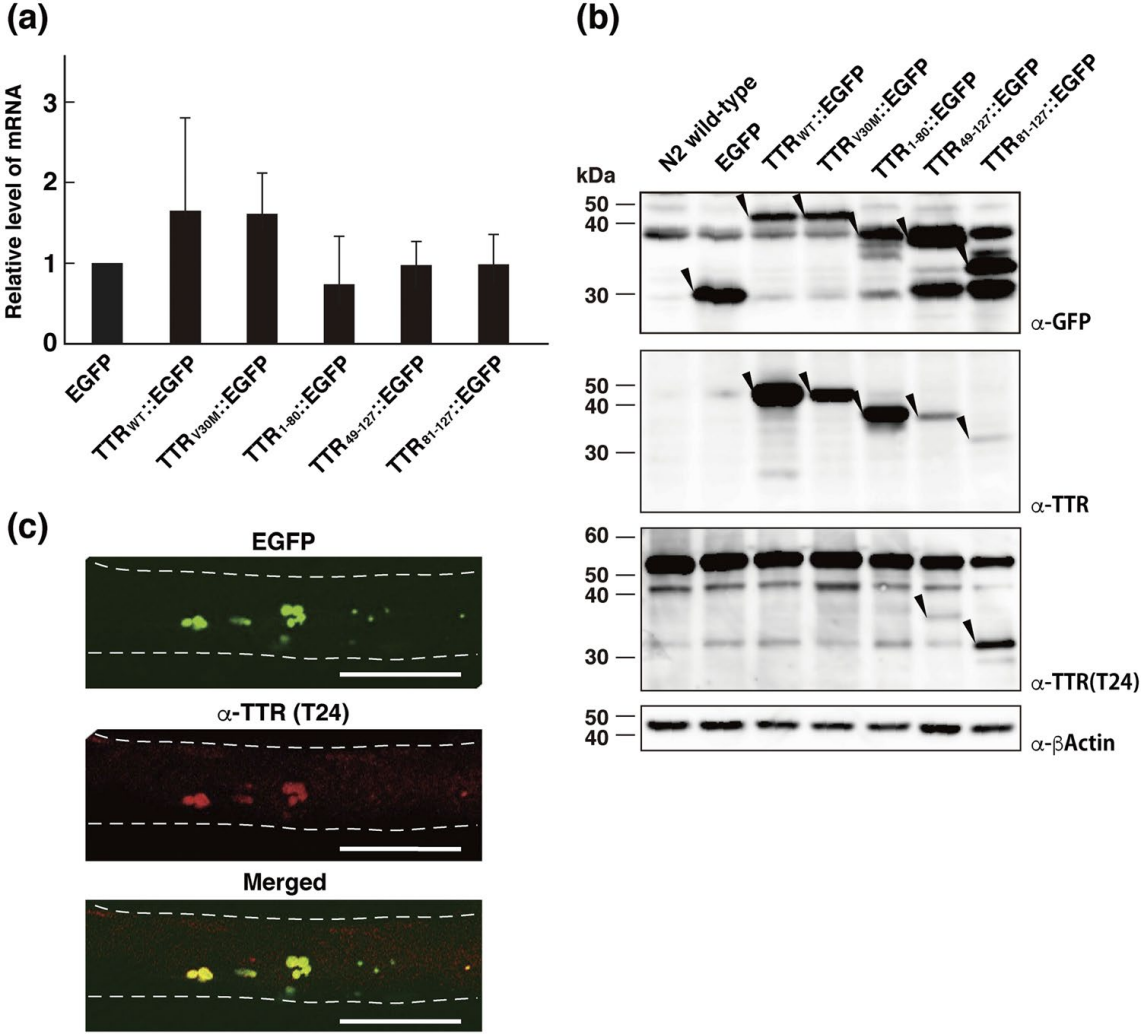
Analyses of the expression of TTR::EGFP fusion proteins in transgenic worms. (**a**) Quantitative RT-PCR analysis was performed to detect transcripts encoding EGFP from six strains of transgenic worms. The transcript levels were normalized by using *act-1* transcripts as internal controls. The relative levels of mRNA were calculated as fold ratios relative to the mRNA level of EGFP-expressing strain. The means and standard deviations from three independent analyses are shown. (**b**) Total lysates from six strains of transgenic worms and the N2 wild-type worm as a control were analyzed by using SDS-PAGE and immunoblotting with the antibodies anti-GFP, polyclonal anti-TTR, monoclonal anti-C-terminal TTR (T24), and anti-β-actin (as a loading control). Molecular masses are indicated at the left of the panels. Arrowheads point to the bands of interest. Full size scans of immunoblots are shown in Supplementary Fig. S1. (**c**) An EGFP fluorescent image (top), immunohistochemical image (middle) obtained with the anti-C-terminal TTR antibody (T24) from a worm expressing TTR_81–127_::EGFP, and their merged image (bottom) are shown. Broken lines outline the worm surface. Scale bars: 100 μm.

To confirm the expression of TTR::EGFP fusion proteins in transgenic animals, we first measured their transcript levels by quantitative RT-PCR with primers for the gene encoding EGFP. As Fig. 2a illustrates, we found no significant differences. We then performed Western blotting analysis by using an anti-TTR polyclonal antibody, an anti-C-terminal TTR monoclonal antibody (T24)^38^ that recognized residues 115–124 containing the β-strand H of TTR, and an anti-GFP antibody (Fig. 2b). The anti-GFP antibody detected the EGFP protein and all TTR::EGFP fusion proteins at the expected molecular sizes, although a significant cleaved product, which seems to be EGFP, was also detected in some TTR::EGFP proteins. The anti-TTR polyclonal antibody detected all TTR::EGFP fusion proteins, although this antibody had a low affinity for C-terminal TTR proteins. TTR_49–127_::EGFP and TTR_81–127_::EGFP, however, were clearly detected by the T24 antibody. These results suggest that these fusion proteins are indeed expressed in transgenic worms.

Next, to confirm whether signals for EGFP and TTR co-exist in aggregates, we studied the TTR_81–127_::EGFP strain by means of fluorescent immunohistochemistry. The TTR signal colocalized with EGFP-containing aggregates in the body wall muscle cells (Fig. 2c), indicating that the fluorescent aggregates contained TTR_81–127_::EGFP fusion proteins.

**Figure 3:**
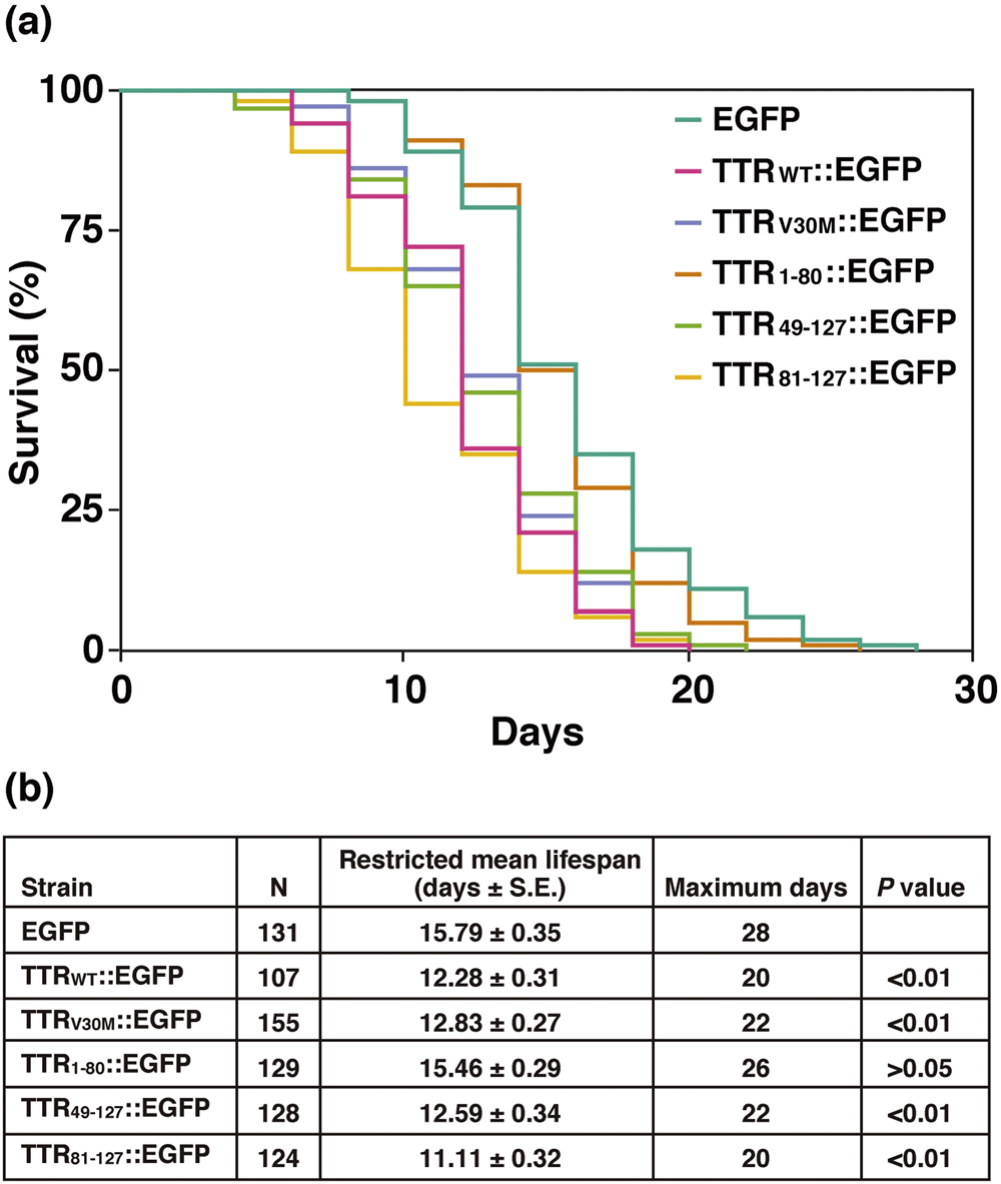
Effect of TTR::EGFP fusion proteins on lifespan. (**a**) More than 100 worms of each of the six kinds of transgenic worms expressing EGFP, TTR_WT_::EGFP, TTR_V30M_::EGFP, TTR_1–80_::EGFP, TTR_49–127_::EGFP, or TTR_81–127_::EGFP were used to investigate worm lifespan. The data were processed via Kaplan–Meir survival analysis of OASIS2 (https://sbi.postech.ac.kr/oasis2/). Plots represent at least two independent experiments. Colour was determined with the aid of the COLORBREWER 2.0 software (www.colorbrewer2.org). (**b**) Restricted mean lifespans, maximum days, and *P* values are shown for the different strains.

Finally, to evaluate the toxicity of TTR::EGFP aggregates in transgenic worms, we measured the lifespan of these worms. Although it is well known that fluorodeoxyuridine (FUdR) has no effect on the lifespan of wild-type N2 worms, it has been reported that FUdR has some effect on the lifespan of some mutants, such as *gas-1*, in an FUdR concentration-dependent manner^39^. Therefore, we used FUdR at a relatively low concentration of 16 μM (compared to the standard 100 μM) in our lifespan assays. Figure 3 shows that the TTR_81–127_::EGFP strain had the shortest lifespan among all transgenic worms. The TTR_1–80_::EGFP strain that rarely formed aggregates had the same lifespan as the EGFP strain. Lifespans of the TTR_WT_::EGFP, TTR_V30M_::EGFP, and TTR_49–127_::EGFP strains fell between these two values. These results indicate that the formation of aggregates may be related to toxicity. On the basis of these data, we expect that transgenic worms forming toxic TTR aggregates may be a suitable animal model of ATTR amyloidosis and can be used to screen drug candidates that would cure and/or prevent amyloidosis.

### EGCG inhibited the formation of TTR::EGFP aggregates *in vivo*

Using our novel transgenic worm models, we investigated the therapeutic effects of three candidate drugs: diflunisal, β-cyclodextrin, and EGCG. Diflunisal stabilizes TTR tetramers and inhibits the progression of amyloidogenesis^8^. β-Cyclodextrin reportedly interacts with TTR and prevents protein misfolding^40^. EGCG is a main active ingredient of green tea and reportedly inhibits protein aggregation and remodels mature amyloid fibrils consisting of Aβ_1–40_, IAPP_8–24_, or Sup35NM_7–16_^41^.

**Figure 4:**
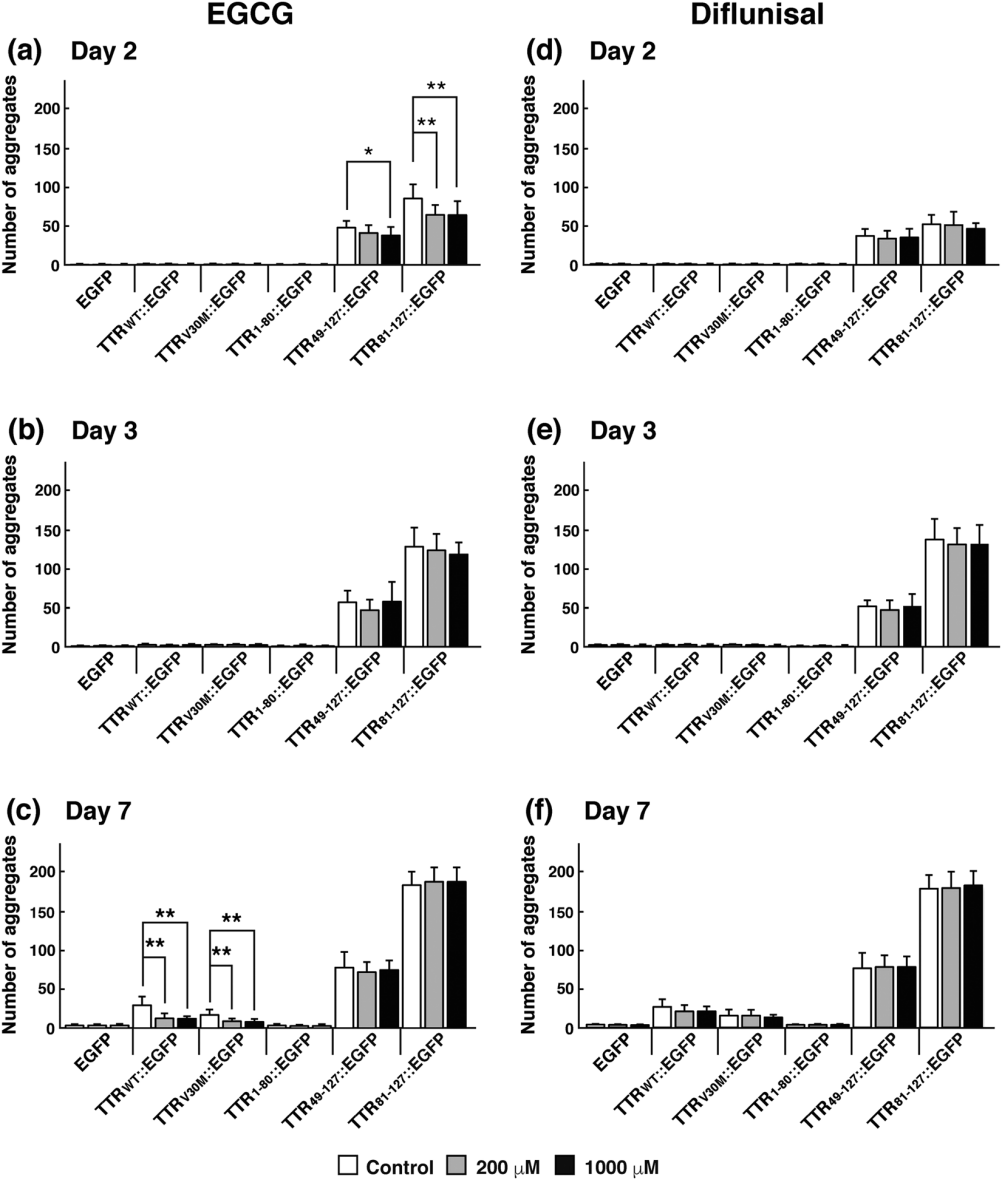
Effects of EGCG and diflunisal on the formation of TTR::EGFP aggregates in transgenic worms. Six kinds of transgenic worms expressing EGFP, TTR_WT_::EGFP, TTR_V30M_::EGFP, TTR_1–80_::EGFP, TTR_49–127_::EGFP, or TTR_81–127_::EGFP were used. Effects of EGCG (**a**–**c**) and diflunisal (**d**–**f**) on aggregation in worms were analyzed, and the number of aggregates in each worm was counted on days 2 (**a**,**d**), 3 (**b**,**e**), and 7 (**c**,**f**). The day when eggs were laid was defined as day 0. Worms were grown in the absence of EGCG (white bars) or in the presence of 200 μM (gray bars) or 1000 μM (black bars) EGCG. Plots represent at least three independent experiments (16 animals for each strain). Error bars indicate the SD. *P < 0.05, **P < 0.01.

To elucidate the effect of these candidate drugs on the formation of aggregates, we counted the number of aggregates in transgenic worms grown in the presence or absence of the drugs. We found that the number of aggregates was reduced in L3 larvae of TTR_49–127_::EGFP and TTR_81–127_::EGFP worms fed EGCG (day 2 in Fig. 4a). Note that we defined the day when eggs were laid as day 0. A higher concentration of EGCG had a stronger effect. However, this inhibitory effect of EGCG on aggregate formation gradually decreased as worms aged (Fig. 4a–c). In contrast, EGCG inhibited TTR aggregate formation in aged worms of TTR_WT_::EGFP and TTR_V30M_::EGFP strains (day 7 in Fig. 4c). Given that the formation of aggregates was observed in earlier developmental stages in TTR_49–127_::EGFP and TTR_81–127_::EGFP worms but not in TTR_WT_::EGFP and TTR_V30M_::EGFP worms, as shown in Fig. 1 and Table 1, these results suggest that EGCG has an inhibitory effect on aggregate formation and delays the onset of aggregate formation. Diflunisal and β-cyclodextrin, however, did not inhibit TTR aggregate formation in the transgenic worms (Fig. 4d–f and Supplementary Fig. S2).

### EGCG improved the motility of transgenic worms

**Figure 5:**
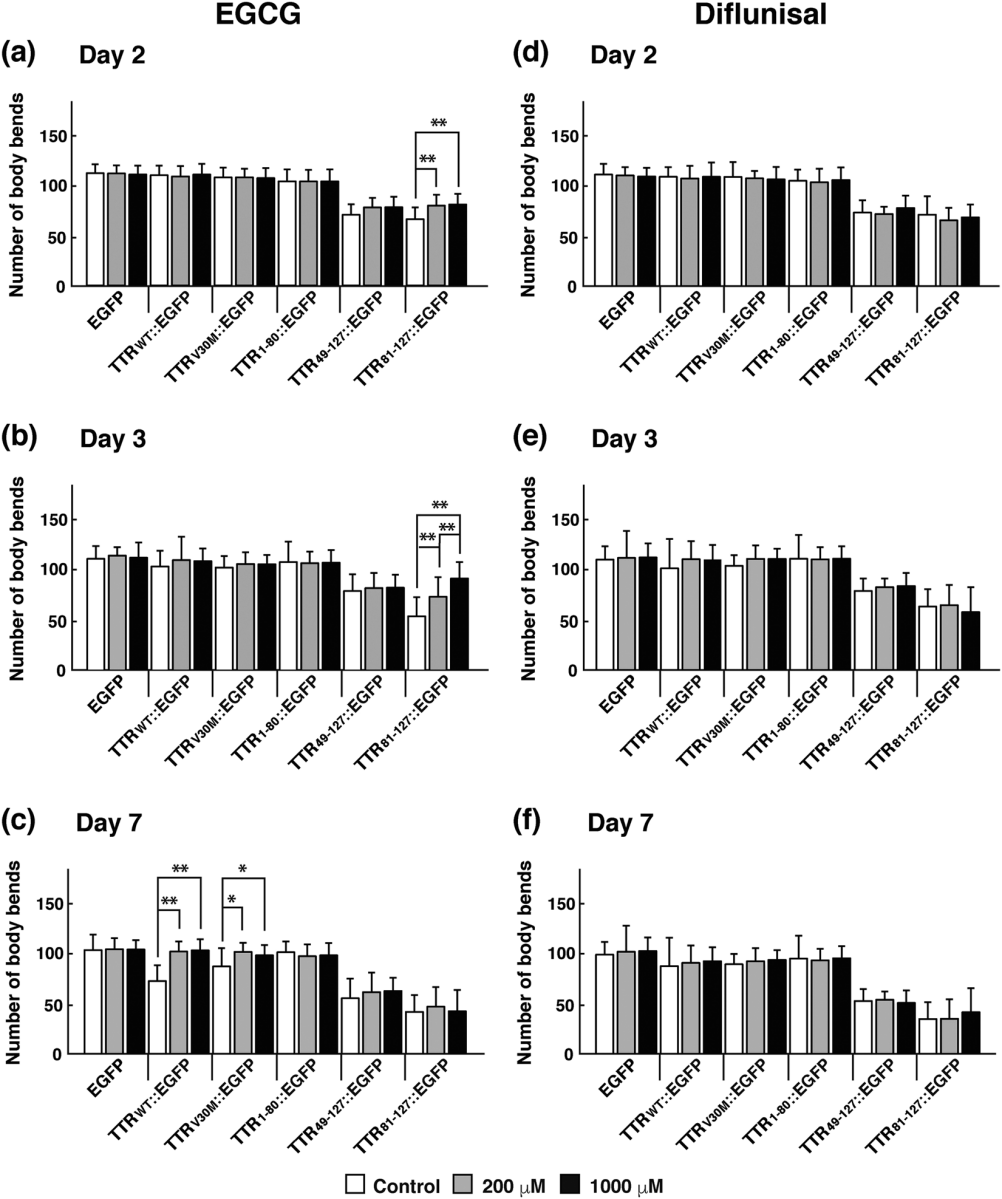
Effects of EGCG and diflunisal on motility of transgenic worms. Six kinds of transgenic worms expressing EGFP, TTR_WT_::EGFP, TTR_V30M_::EGFP, TTR_1–80_::EGFP, TTR_49–127_::EGFP, or TTR_81–127_::EGFP were used. Effects of EGCG (**a**–**c**) and diflunisal (**d**–**f**) on worm motility were analyzed, and the number of body bends of worms per minute was counted in M9 buffer on days 2 (**a**,**d**), 3 (**b**,**e**), and 7 (**c**,**f**). The day when eggs were laid was defined as day 0. Worms were grown in the absence of EGCG (white bars) or in the presence of EGCG at 200 μM (gray bars) or 1000 μM (black bars). Plots represent at least three independent experiments (16 animals for each strain). Error bars indicate SD. *P < 0.05, **P < 0.01.

As Fig. 5 illustrates, TTR_49–127_::EGFP and TTR_81–127_::EGFP worms manifested a severe motility defect from the larval stage. Given that these two transgenic strains had clear and discrete aggregates as seen in Fig. 1, these results indicate that TTR::EGFP aggregates demonstrated toxicity *in vivo*. We next evaluated the motility of transgenic worms in the presence and absence of three candidate drugs at different developmental stages. Worms were placed in M9 buffer, and the number of body bends of the worms was counted. We found that EGCG improved the motility of larvae of TTR_49–127_::EGFP and TTR_81–127_::EGFP worms but not that of the aged worms (Fig. 5a–c). EGCG did improve the motility of aged TTR_WT_::EGFP and TTR_V30M_::EGFP worms, however (Fig. 5c). As in the case of the effects of diflunisal and β-cyclodextrin on aggregate formation, these drugs had no effect on motility in any worm strain (Fig. 5d–f and Supplementary Fig. S3). Taken together with the effects on aggregate formation, only EGCG among the three drugs had inhibitory activity against toxic TTR::EGFP aggregates.

### EGCG improved the survival of transgenic worms

**Figure 6:**
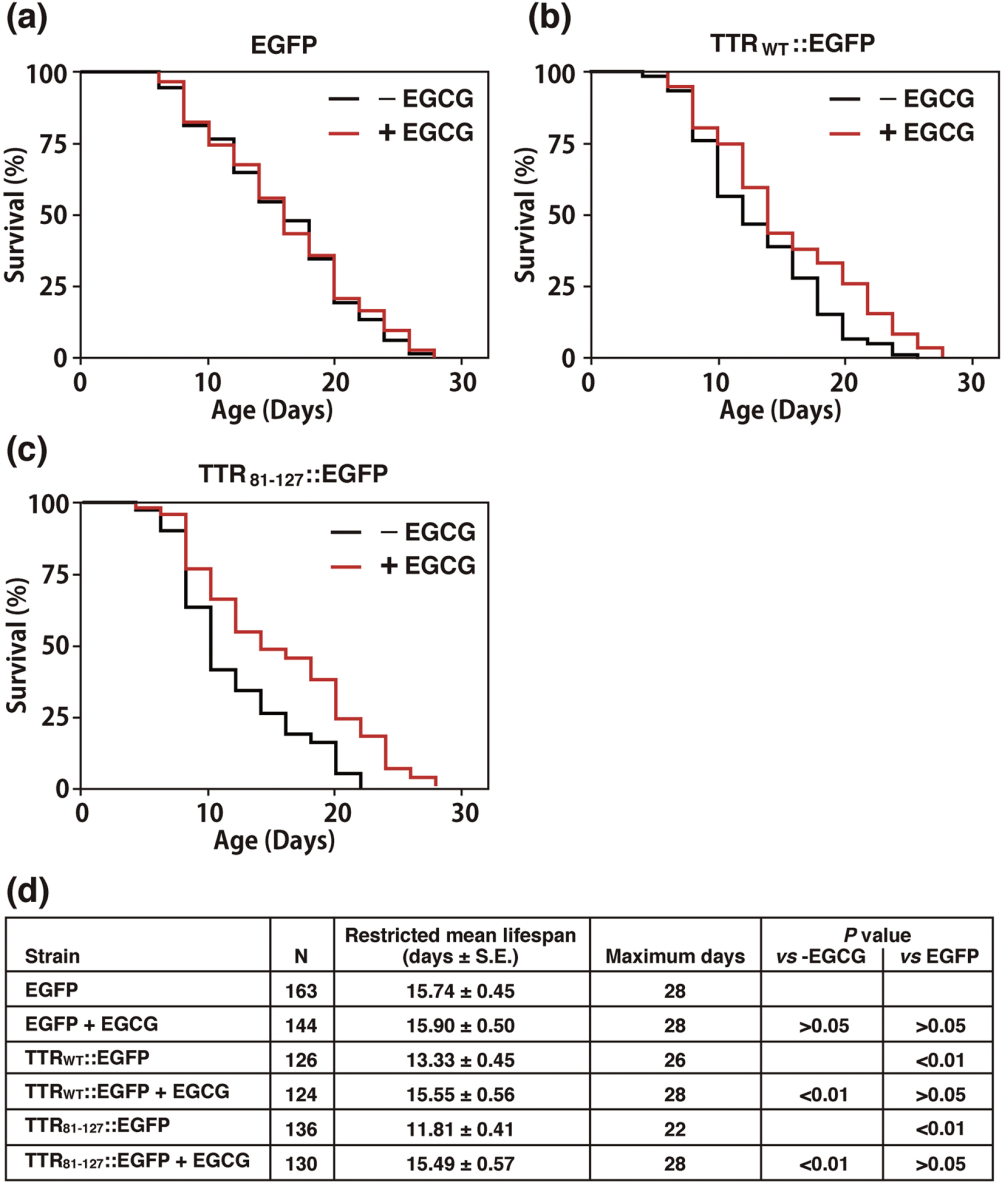
Effect of EGCG on lifespan of transgenic worms. Three kinds of transgenic worms expressing EGFP (**a**), TTR_WT_::EGFP (**b**), or TTR_81–127_::EGFP (**c**) were studied in the absence (black line) and presence (magenta line) of 1 mM EGCG. Data were processed via Kaplan–Meir survival analysis of OASIS 2 (https://sbi.postech.ac.kr/oasis2/). Plots represent at least two independent experiments. (**d**) Restricted mean lifespans, maximum days, and *P* values are shown.

To determine whether EGCG can improve survival of transgenic worms, we used three transgenic strains, the EGFP alone strain as a control, the TTR_WT_::EGFP strain showing a moderately shortened lifespan, and the TTR_81–127_::EGFP strain showing a severely shortened lifespan (Fig. 3). We found that EGCG had no effect on EGFP-expressing controls (Fig. 6a,d), but it significantly improved the lifespans of animals expressing either TTR_WT_::EGFP or TTR_81–127_::EGFP (Fig. 6b–d).

### EGCG reduced the intracellular ROS level in the worm model

**Figure 7:**
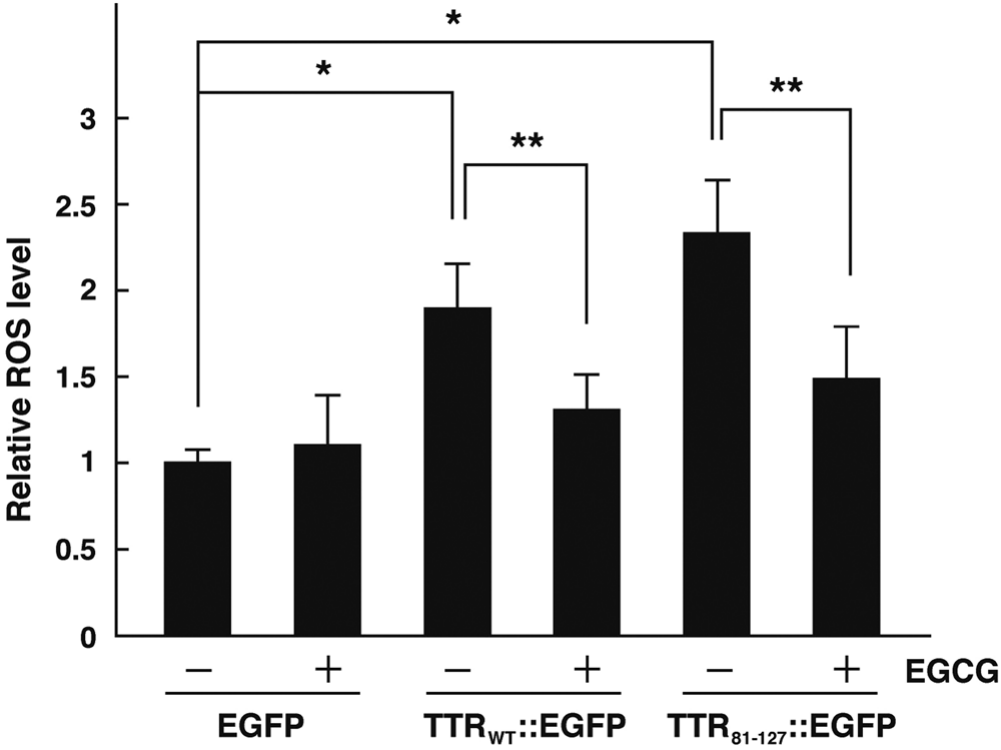
Effect of EGCG on ROS levels in transgenic worms. Three kinds of transgenic worms expressing EGFP, TTR_WT_::EGFP, or TTR_81–127_::EGFP were used. The synchronized L1 worms were grown on NGM agar plates in the absence or presence of 1 mM EGCG for 3 days. ROS levels were measured with H2DCF-DA as a molecular probe. Results are expressed as relative ROS levels (relative to the untreated EGFP worms). Error bars indicate SD. *P < 0.05, **P < 0.01.

One interesting finding was that EGCG significantly restored the shortened lifespan of worms expressing TTR_81–127_::EGFP but did not inhibit aggregate formation or improve the motility defect in aged worms expressing TTR_81–127_::EGFP. These results suggested that EGCG may have a therapeutic effect on the model worms other than inhibiting aggregate formation. A previous study reported a relationship between the pathogenesis of ATTR amyloidosis and reactive oxygen species (ROS) in TTR amyloid deposits in the tissues of patients^42^. EGCG also reportedly possesses free radical-scavenging activity^41,43^. Therefore, we measured cellular ROS levels in transgenic worms in the presence and absence of EGCG by using 2′,7′-dichlorodihydrofluorescein diacetate as a molecular probe. The TTR_WT_::EGFP and TTR_81–127_::EGFP strains showed higher ROS levels than did control worms, which suggests that TTR aggregates increased the cellular ROS levels in *C*. *elegans*. In the presence of EGCG, however, the higher ROS levels in the two TTR_WT_::EGFP and TTR_81–127_::EGFP strains were reduced to nearly the control level (Fig. 7). These results clearly indicate that TTR aggregates increased cellular ROS levels and that EGCG demonstrated free radical-scavenging activity in *C*. *elegans*.

## Discussion

In this study, we generated five kinds of transgenic *C*. *elegans* strains and established that the C-terminal fragments of TTR, TTR_49–127_, and TTR_81–127_ easily formed aggregates *in vivo*. TTR_49–127_ has been found in amyloid deposits of patients^12–15^. TTR_49–127_ and TTR_81–127_ contain two β-strands F and H (residues 91–96 and 115–124, respectively), both of which have been shown to form amyloid efficiently *in vitro*^18^. These results suggest that the C-terminal fragments of TTR containing the amyloidogenic β-strands are prone to form aggregates *in vivo*, a finding that supports the hypothesis that the C-terminal fragment of TTR plays an important role in amyloidogenesis in tissues of patients with ATTR amyloidosis. Furthermore, transgenic worms expressing TTR_49–127_::EGFP or TTR_81–127_::EGFP evidenced reduced motility and a shortened lifespan, results that indicate that TTR aggregates contributed to cellular toxicity *in vivo*. In view of the findings that TTR_1–80_::EGFP did not produce aggregates or cause cellular toxicity *in vivo* and that cellular toxicity in the TTR_81–127_::EGFP strain was stronger than that in the TTR_49–127_::EGFP strain, the TTR_81–127_ fragment may be critical for toxicity. The TTR_WT_::EGFP and TTR_V30M_::EGFP strains showed mild toxicity as the worms aged, however. The TTR_49–127_ fragment may be generated by proteolytic cleavage in patients^16^. C-terminal fragments may thus be produced and accumulate as worms age. Additional *in vivo* studies are required to determine how the C-terminal TTR fragment is involved in the pathogenesis of TTR amyloidosis.

In view of the cellular toxicity of TTR aggregates, our *C*. *elegans* model has potential as a screening tool for therapeutic agents for ATTR amyloidosis. In this study, we evaluated the effects of three candidate drugs on the *C*. *elegans* model. We found that when model worms were treated with EGCG, the accumulation of TTR aggregates and the motility defect were delayed, and the lifespan was improved. Note that EGCG did not disrupt or dissociate the aggregates formed, however. Our *in vivo* observation is supported by the fact that the binding of EGCG to TTR modulates the aggregation mechanism of TTR^44^. EGCG also reportedly reduced Aβ-induced and ataxin-3-induced toxicity in transgenic worm models^33,36,45^. These results suggest that EGCG has an inhibitory effect against aggregate-induced cellular toxicity. We thus propose that EGCG combined with a putative aggregate disruptor may be a powerful therapeutic strategy. Alternatively, by using EGCG as a lead compound, a new compound with aggregate disruptor activity may be developed. Our *C*. *elegans* model will aid such a process.

Diflunisal and β-cyclodextrin, however, had no therapeutic effect in our transgenic worms. Diflunisal as a TTR tetramer stabilizer may not have a therapeutic effect on the intracellular monomeric TTR species in our model worms, in which TTR::EGFP proteins are produced and exist in the cytoplasm. Very recently, Madhivanan *et al*. reported that non-tagged, secreted TTR_V30M_ can form non-native oligomers in the body cavity, causing defects in neuronal morphology and locomotion^46^. Interestingly, CMPD5, a TTR-selective fluorogenic dye, binds the TTR tetramer in the body cavity and functions as a tetramer stabilizer. Therefore, CMPD5 feeding rescued the locomotion defect caused by TTR_V30M_^46^. On the other hand, it can be reasonably assumed that β-cyclodextrin does not inhibit TTR misfolding sufficiently because of its poor intestinal adsorption^47^. The worm is also covered with a robust cuticle that forms a barrier to chemical uptake. It is interesting to mention that several mutations, including *bus-5(br19)* with altered cuticle properties, have been identified, and these mutants showed increased permeability, such that they can be used as a suitable strain for drug screening^48^.

TTR is mainly synthesized by the liver for secretion into the blood, and it usually exists as a tetramer^4^. In patients, TTR with mutation tends to dissociate and form amyloid fibrils^5^. Therefore, although we generated the *C*. *elegans* model system in body wall muscle cells for the TTR study, we still need to establish a secretion system of TTR_WT_, TTR_V30M_, and TTR_81–127_ and a corresponding detection system in *C*. *elegans* to fully understand the mechanism of TTR amyloidosis. Previously, it has been reported that TTR_WT_ was expressed in body wall muscle cells and secreted (strain CL2008). Although secreted TTR was detected throughout the animal, with an intense signal in coelomocytes using an anti-TTR antibody, thioflavin S (an amyloid specific dye)-positive deposits were not observed^49^. Later, those TTR-expressing coelomocytes in CL2008 were detected using the newly developed amyloid-specific dye X-34^50^. The newly developed TTR-selective fluorogenic probe, aryl fluorosulfate 4, can also detect secreted TTR tetramers^51^. Using these probes, it will be possible to discriminate native-TTR (tetramer) and non-native-TTR (oligomer and amyloid) in our future secretion system. In the present study, TTR_81–127_ was likely to form intracellular amorphous shape aggregates, but we have not determined detailed morphological features of these aggregates. To elucidate detailed morphological features of these TTR aggregates, we should perform further ultrastructural investigations. In addition, it is important to decipher this issue in the future secretion model.

We also demonstrated that TTR aggregates increased the cellular ROS level in transgenic worms and that EGCG inhibited the increase in the cellular ROS level in aggregate-forming transgenic worms. A high ROS level has been proposed to be one of the primary causes of aging in *C*. *elegans*^52^. We thus conclude from these data that EGCG possesses two activities—inhibition of TTR aggregate formation and reduction of accumulated intracellular ROS levels. EGCG may be a good lead compound to develop an effective therapeutic drug for amyloidosis, and the *C*. *elegans* model system is ideal for such a screening process. It is important to mention that the TTR_WT_::EGFP strain at day 3 presented a higher ROS level (Fig. 7) but lower level of aggregates (Fig. 4) and body bends impairment (Fig. 5). This can be explained as follows: it takes time to form TTR_WT_::EGFP aggregates after synthesis. During this process, a soluble monomer becomes a soluble oligomer, and then becomes soluble and insoluble aggregates. When the soluble oligomer is formed, the ROS level may increase, but the phenotypic impairment may not yet appear. It is known that TTR is frequently and post-translationally modified by oxidation. When the TTR-expressing strain CL2008 was treated with oxidative stress-inducing reagents, such as menadione, modified TTR was formed, causing the toxic effect. However, when an anti-oxidant was treated simultaneously, the toxic effect was not observed, suggesting that the *C*. *elegans* model system is suitable for the post-translational protein modification analysis^53^.

In conclusion, our newly developed *C*. *elegans* model will be useful for *in vivo* pathological analyses of TTR amyloidosis as well as drug screening.

## Methods

### Chemicals

EGCG (Tokyo Chemical Industry, Tokyo, Japan) and β-cyclodextrin (a gift from Dr. H. Jono, Department of Clinical Pharmaceutical Sciences, Kumamoto University) were dissolved in water and stored at −20 °C. Diflunisal (Sigma-Aldrich, St. Louis, MO, USA) was dissolved in 100% dimethyl sulfoxide (DMSO) and stored at −20 °C. Drugs were added after pouring plates. The control group was treated with the solvent used, minus the drug (i.e. add water for EGCG and β-cyclodextrin controls), and the equivalent concentration (a final concentration of 0.05%) of DMSO was used for the diflunisal control.

### Plasmid construction

To create a translational fusion of human full-length TTR with an EGFP, the full-length human cDNA for TTR was amplified via PCR by using PrimeStar HS DNA polymerase (TaKaRa, Otsu, Japan) and the primers ns-fTTR-5ʹ-*Sal*I and TTR-3ʹ-*Bam*HI and was cloned into pEGFP-1 (Clonetech, Palo Alto, CA, USA) to yield pCKX3305. A resultant fusion gene for TTR::EGFP was then amplified with the primers ns-fTTR-5ʹ-*Sal*I and EGFP-3ʹ-*Eco*RV and cloned into pPD30.38 containing the promoter and enhancer elements from *unc-54* myosin heavy-chain locus^54^ to yield pCKX3307. The fusion protein will be expressed in *C*. *elegans* body wall muscle cells. To create a translational fusion of a truncated TTR_81–127_ with EGFP, we amplified a DNA fragment for TTR_81–127_ with the primers TTR-5ʹ-*Sal*I and TTR-3ʹ-*Bam*HI and cloned it into pEGFP-1 to yield pCKX3301. We then amplified TTR_81–127_::EGFP with pCKX3301 as a template and the primers TTR-5ʹ-*Sal*I and EGFP-3ʹ-*Eco*RV and cloned it into pPD30.38 to yield pCKX3303. To create other translational fusions of truncated human TTR with EGFP, we performed PCR with pCKX3307 as a template and the primer sets 1–80-down and 1–80-up for TTR_1–80_::EGFP, and ns49–127-down and ns49–127-up for TTR_49–127_::EGFP, by using the QuikChange II Site-Directed Mutagenesis Kit (Agilent Technologies, Cedar Creek, TX, USA), to yield pCKX3311 and pCKX3313, respectively. To create a point mutation for V30M, we performed PCR with pCKX3307 as a template and the primers V30M-down and V30M-up, by using the QuikChange II Site-Directed Mutagenesis Kit, to yield pCKX3309. To create an EGFP expression vector, we performed PCR with pCKX3307 as a template and the primers nsEGFP-down and nsEGFP-up, by using the QuikChange II Site-Directed Mutagenesis Kit, to yield pCKX3316.

Plasmid DNA was column purified by using the Wizard Plus SV Minipreps DNA Purification system (Promega, Madison, WI, USA) and filtered by using SURPEC-02 (TaKaRa). DNA sequences of the constructed plasmids were verified by DNA sequencing. Supplementary Table S1 summarizes the oligonucleotide primers used in this study.

### *C. elegans* strains and protocols

*C*. *elegans* N2 (Bristol) was used as the wild-type strain. Standard nematode growth medium (NGM) was used for *C*. *elegans* growth and maintenance at 20 °C^55^. NGM agar plates were seeded with live *Escherichia coli* OP50.

Transgenic animals carrying extrachromosomal arrays were created by microinjecting the DNA plasmid into the gonad of young adults at a concentration of 0.1 mg/ml. Individual fluorescent F_2_ worms were isolated to establish transgenic lines. At least two independent lines for each construct were isolated and analyzed.

### Body bends assays

Body bends assays were performed as described previously^56^. Worms were placed in M9 buffer, and the number of body bends of the worms was counted manually for 1 min when a worm swung its head to either left or right. Approximately 15 worms were analyzed for each experimental condition. Worms were analyzed at days 2, 3, and 7. The day when eggs were laid was defined as day 0.

### Lifespan assays

Lifespan assays were performed essentially according to standard protocols as described in Murayama *et al*.^57^. Briefly, synchronized young adults were treated with 0.3 mg/ml fluorodeoxyuridine (FUdR) for 24 h to prevent production of progeny. The worms were then transferred to fresh 0.2 mg/ml (16 μM) FUdR-containing plates in the presence or absence of EGCG (final concentration 1 mM). Surviving and dead worms were counted every other day. We did not count worms that died due to internal hatching or crawling up the plate wall. The day when worms reached the young adult stage was said to be day 0. Lifespan assays were repeated at least twice. Data analysis was performed as described by Han *et al*.^58^, by using the publicly available analysis suite OASIS 2 (Online Application for Survival Analysis 2) (https://sbi.postech.ac.kr/oasis2/).

### Fluorescence microscopic observation

Worms were placed in M9 buffer containing levamisole (0.5 mg/ml) on a slide glass, and images were obtained via the Olympus Power BX51 microscope equipped with a CoolSnapHQ CCD camera. MetaMorph software was used to process acquired images.

Worms were placed in M9 buffer containing levamisole (0.5 mg/ml) on a slide glass, and the number of TTR::EGFP aggregates in body wall muscle cells was counted manually via the Olympus SZX16 fluorescence microscope with a x115 magnification. Aggregates were defined as discrete structures with spherical shape and boundaries easily distinguishable from surrounding fluorescence on all sides. Approximately 10 worms were analyzed for each experimental condition. Worms were evaluated at days 2, 3, and 7. The day when eggs were laid was said to be day 0.

### Fluorescence immunohistochemistry

TTR_81–127_::EGFP-expressing transgenic worms were fixed in Bouin’s fixative/methanol/β-mercaptoethanol (40:40:1) and immunostaining was performed^59,60^. The primary and secondary antibodies used were the mouse anti-TTR monoclonal antibody (T24) at a 50× dilution and the goat anti-mouse IgG H&L (Alexa Fluor 594) antibody at a 50× dilution, respectively. A confocal laser scanning microscope (FV1200; Olympus, Tokyo, Japan) was used to visualize stained samples.

### Quantitative real-time PCR for mRNA quantification

L4-stage worms of each strain grown on 6-cm NGM plates were collected into 1.5-ml tubes and washed three times with M9 buffer (22 mM KH_2_PO_4_, 42 mM Na_2_HPO_4_, 85.5 mM NaCl, 1 mM MgSO_4_) containing 0.01% Tween 20. Total RNA was extracted with TRIzol (Life Technologies, Carlsbad, CA, USA) and reverse-transcribed to cDNA by using an ExScript RT reagent kit (TaKaRa) according to the manufacturer’s instructions. The LightCycler System (Roche Diagnostic, Basel, Switzerland) with SYBR Premix DimerEraser (TaKaRa) was used to perform all PCR reactions with primers of EGFP forward and EGFP reverse. β-Actin was used as an internal control with primers of β-actin forward and β-actin reverse. Supplementary Table S1 summarizes the oligonucleotide primers used in this study.

### Western blotting

L4-stage worms of each strain grown on 6-cm NGM plates were collected into 1.5-ml tubes and washed three times with M9 buffer containing 0.01% Tween 20. The worm pellets were resuspended in 100 μl of PBS and frozen in liquid nitrogen until use. Total lysates were prepared by sonication, and protein concentration was determined by using a BCA kit (Thermo Fisher Scientific, Waltham, MA, USA). Samples were mixed with SDS sample buffer and analyzed by SDS-PAGE by using 4–20% Mini-PROTEAN TGX Precast Protein Gels (Bio-Rad Laboratories, Hercules, CA, USA) or 12% SDS-PAGE. The TTR::EGFP protein species in the samples were detected via standard Western blotting protocol by using an anti-TTR antibody (A0002, polyclonal rabbit anti-human prealbumin; DAKO, Copenhagen, Denmark) (1:1000), the monoclonal antibody T24 (1:100) that recognizes residues 115–124 (cryptic epitope) containing the β-strand H of TTR as described previously^38^, an anti-GFP monoclonal antibody (1:2000) (Clonetech), and an anti-β-actin monoclonal antibody (1:3000) (Sigma-Aldrich). β-Actin was used as a loading control. HRP-conjugated goat anti-rabbit immunoglobulins antibody (DAKO) (1:1000) and HRP-conjugated rabbit anti-mouse immunoglobulins antibody (DAKO) (1:1000) were used as a secondary antibody for the anti-TTR antibody and other antibodies, respectively. Signals were detected using the ECL Prime Western Blotting Detection Reagent (GE Healthcare, Pittsburgh, PA, USA) and LAS-4000 Image Analyser (GE Healthcare).

### Measurement of ROS

Intracellular ROS levels in *C*. *elegans* were measured with 2′,7′-dichlorodihydrofluorescein diacetate (H2DCF-DA) as a molecular probe. The hatched worms were grown on NGM plates in the presence or absence of 1 mM EGCG for 3 days, after which they were collected and resuspended in 100 μl of PBS containing 0.01% Tween 20. Worms were sonicated, and the lysates obtained were incubated with H2DCF-DA (50 mM final concentration) in a Costar 96-well microtiter plate at 37 °C. Samples were read every 15 min for 2 h with an xMark Microplate Spectrophotometer (Bio-Rad Laboratories) with the emission wavelength at 530 nm. Assays were repeated three times.

### Statistical analysis

Statistical significance for the number of aggregates, the number of body bends, and ROS levels was analyzed by a Steel-Dwass’s multiple comparison post-hoc test using JMP 9.0 software (SAS Institute Japan, Tokyo, Japan). For all analyses, a *P* value of <0.05 was considered statistically significant.

## Electronic supplementary material

Supplementary Information

## Data Availability

All data generated or analyzed during this study are included in this published article and its Supplementary Information Files.
