# Supplementary material for: Development of transgenic Caenorhabditis elegans expressing human transthyretin as a model for drug screening
Source: Sci Rep. 2018 Dec 14;8:17884. doi: 10.1038/s41598-018-36357-5 (PMC6294829; doi:10.1038/s41598-018-36357-5)
Supplement: Supplementary file 1 — Supplementary Information [file 41598_2018_36357_MOESM1_ESM.pdf]

Supplementary Information

Development of transgenic *Caenorhabditis elegans* expressing human transthyretin as a model for drug screening

Yukimoto Tsuda[1], Kunitoshi Yamanaka[2]*, Risa Toyashima[1], Mitsuharu Ueda[1]*, Teruaki Masuda[1], Yohei Misumi[1], Teru Ogura[2], & Yukio Ando[1]

[1] Department of Neurology, Graduate School of Medical Sciences, Kumamoto University, 1-1-1 Honjo, Chuo-ku, Kumamoto 860-8556, Japan

[2] Department of Molecular Cell Biology, Institute of Molecular Embryology and Genetics, Kumamoto University, 2-2-1 Honjo, Chuo-ku, Kumamoto 860-0811, Japan

*Correspondence should be addressed to K.Y. (email: yamanaka@gpo.kumamoto-u.ac.jp) or M.U. (email: mitt@rb3.so-net.ne.jp).

Tsuda et al. Supplementary Figure S1

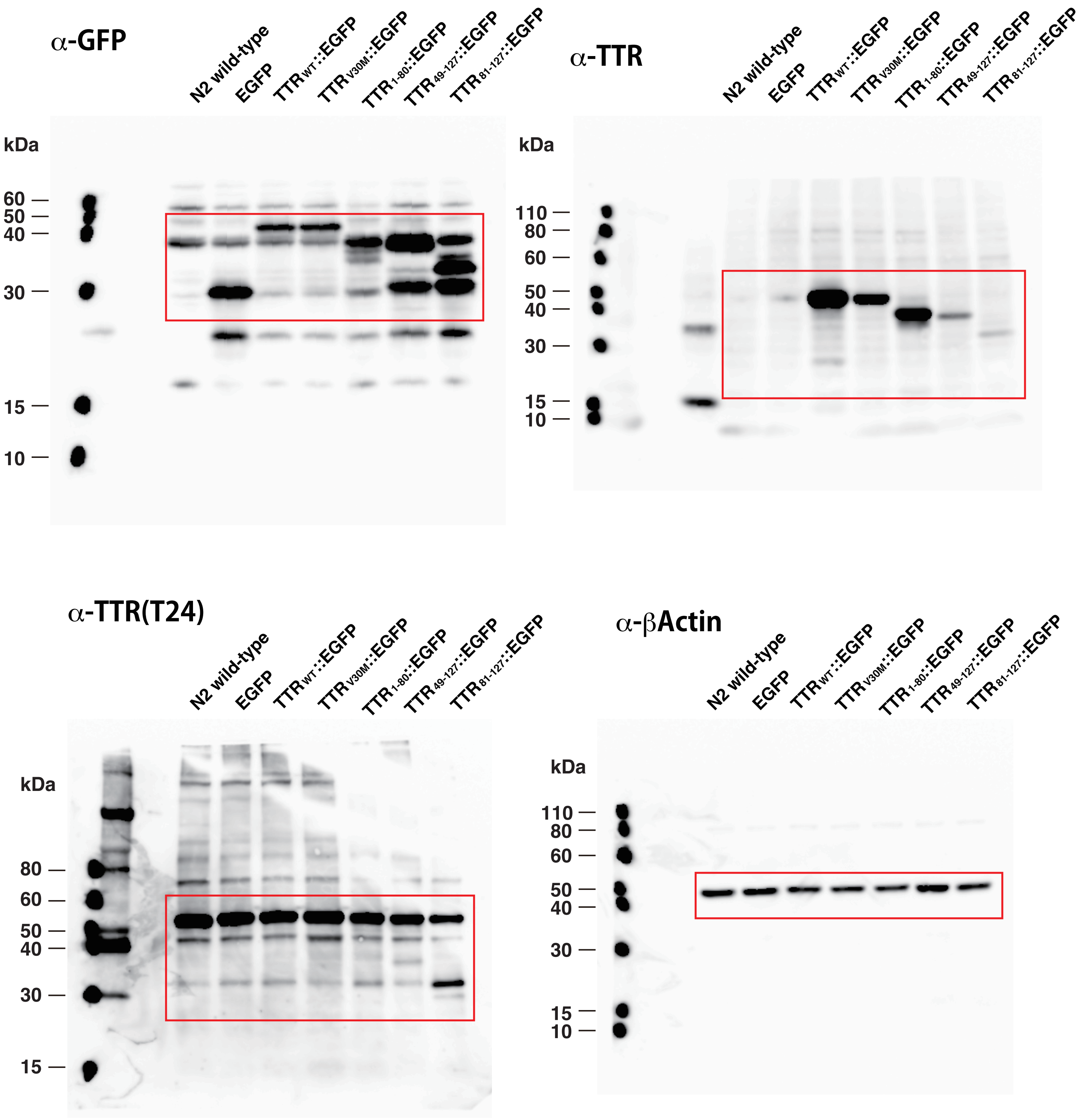

Supplementary Figure S1. Full blots for detection of EGFP, TTR, C-terminal fragments of TTR and β-actin. Portions (red squares) are used in Figure 1b. The positions of molecular markers are shown to the left of the blots.

Tsuda et al. Supplementary Figure S2

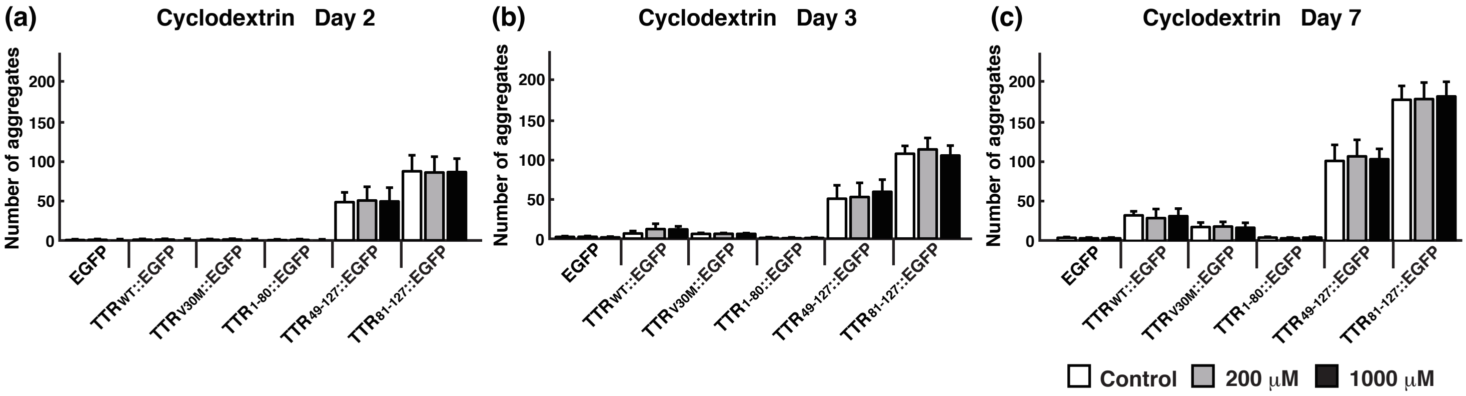

Supplementary Figure S2. Effects of β-cyclodextrin on the TTR::EGFP aggregate formation in transgenic worms. Six kinds of transgenic worms expressing EGFP, TTR_{WT}::EGFP, TTR_{V30M}::EGFP, TTR_{1-80}::EGFP, TTR_{49-127}::EGFP, or TTR_{81-129}::EGFP were used. The number of aggregates in a worm was counted on days 2 (**a**), 3 (**b**) and 7 (**c**). The day when eggs were laid was defined as day 0. Worms were grown in the absence of drug (white bars) or in the presence of the drug at 200 μM (gray bars) or 1000 μM (black bars). Plots represent of at least three independent experiments (16 animals for each strain). Error bars indicate SD.

Tsuda et al. Supplementary Figure S3

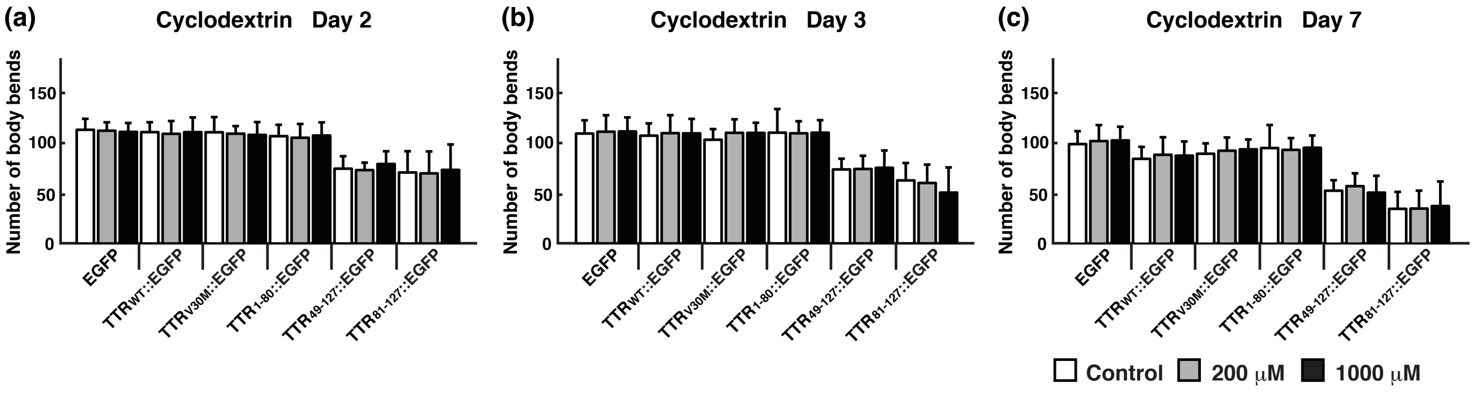

Supplementary Figure S3. Effects of β-cyclodextrin on motility of transgenic worms. Six kinds of transgenic worms expressing EGFP, TTR_{WT}::EGFP, TTR_{V30M}::EGFP, $TTR_{1\text{-}80}$::EGFP, $TTR_{49\text{-}127}$::EGFP, or $TTR_{81\text{-}129}$::EGFP were used. The number of body bends of worms per minute was counted in M9 buffer on days 2 (**a**), 3 (**b**) and 7 (**c**). The day when eggs were laid was said to be day 0. Worms were grown in the absence of drug (white bars) or in the presence of drug at 200 μM (gray bars) or 1000 μM (black bars). Plots represent at least three independent experiments (16 animals for each strain). Error bars indicate SD.

Supplementary Table S1. Oligonucleotide primers used in this study.

**Primer name**	**Sequence (5' to 3')**
TTR-5′-*Sal*I	CCTGGTCGACGCCACCATGGCTTCTCATCGTCTGCTCC
TTR-3′-*Bam*HI	CCAGGGATCCCGTTCCTTGGGATTGGTGACGAC
EGFP-3′-*Eco*RV	CCTGGATATCCGCTTTACTTGTACAGCTCG
ns-fTTR-5′-*Sal*I	CCTGGTCGACGCCACCATGGGCCCTACGGGCACCGGTGAATCC
V30M-down	CCTGCCATCAATGTGGCCATGCATGTGTTCAGAAAGG
V30M-up	CCTTTCTGAACACATGCATGGCCACATTGATGGCAGG
1-80-down	CCAAATCTTACTGGAAGCGGGATCCACCGGTCGC
1-80-up	GCGACCGGTGGATCCCGCTTCCAGTAAGATTTGG
ns49-127-down	GCGTCGACGCCACCATGACCAGTGAGTCTGGAGAG
ns49-127-up	CTCTCCAGACTCACTGGTCATGGTGGCGTCGACGC
nsEGFP-up	CTAGCGTCGACGCCACCATGGTGAGCAAGGGCGAGGAGCTG
nsEGFP-down	CAGCTCCTCGCCCTTGCTCACCATGGTGGCGTCGACGCTAG
EGFP forward	ACGTAAACGGCCACAAGTTC
EGFP reverse	AAGTCGTGCTGCTTCATGTG
β-Actin forward	GTGTGACGACGAGGTTGCCGCTCTTGTTGTAGAC
β-Actin reverse	GGTAAGGATCTTCATGAGGTAATCAGTAAGATCAC
